# Decreased antithrombin activity in the early phase of trauma is strongly associated with extravascular leakage, but not with antithrombin consumption: a prospective observational study

**DOI:** 10.1186/s12959-018-0171-7

**Published:** 2018-08-01

**Authors:** Hironori Matsumoto, Jun Takeba, Kensuke Umakoshi, Satoshi Kikuchi, Muneaki Ohshita, Suguru Annen, Naoki Moriyama, Yuki Nakabayashi, Norio Sato, Mayuki Aibiki

**Affiliations:** Department of Emergency and Critical Care Medicine, Ehime University, Graduate School of Medicine, Shitsukawa 454, Toon City, Ehime 791-0295 Japan

**Keywords:** Albumin, Antithrombin, Coagulofibrinolysis, Consumption coagulopathy, Extravascular leakage, Thrombin activation, Trauma induced coagulopathy

## Abstract

**Background:**

We conducted a prospective observational study for investigating coagulofibrinolytic changes and mechanisms of antithrombin (AT) alternations in trauma.

**Methods:**

Trauma patients hospitalized for more than seven days were analyzed for coagulofibrinolytic biomarkers. The patients were stratified into two groups according to AT activity level on admission (day 0), comprising normal AT and low AT patients.

**Results:**

Thirty-nine patients (median Injury Severity Score 20) exhibited initial coagulatory activation and triphasic fibrinolytic changes. AT activity did not show a negative linear correlation with levels of thrombin-antithrombin complex (TAT), a marker of coagulation activity and AT consumption, but was strongly correlated with levels of albumin (Alb), an index of vascular permeability, on day 0 (*r* = 0.702, *p* <  0.001). Furthermore, Alb was one of the independent predictors for AT on day 0. IL-6 on day 0 and thrombomodulin (TM) levels during the study period, reflecting systemic inflammation and endothelial cell injury, respectively, were significantly higher in the lower AT group (*n* = 10) than in the normal group (*n* = 29) (IL-6, *p* = 0.004; TM, *p* = 0.017). On days 2 and 4, TAT levels in the lower AT group were significantly higher than in the normal group.

**Conclusions:**

Trauma caused clear triphasic coagulofibrinolytic changes. Decreased AT in the later phase might lead to a prolonged hypercoagulation. AT reduction in the initial phase of trauma is strongly associated with extravascular leakage as suggested by the association of Alb depletion with IL-6 and TM elevation, but not with AT consumption.

## Background

Trauma is well known to induce dynamic coagulofibrinolytic changes, which increase bleeding tendency in the initial phase of trauma when the hemostasis becomes uncontrollable [1, 2]. Coagulofibrinolytic disorder has been termed trauma-induced coagulopathy (TIC). The mechanisms of this disorder are still controversial, though they may include disseminated intravascular coagulation (DIC) with a fibrinolytic phenotype or acute traumatic coagulopathy (ATC) [3–5].

The pathophysiology of DIC in the early phase of trauma consists of coagulation activation, hyperfibrinolysis and consumption coagulopathy. Tissue injury due to trauma leads to systemic coagulation activation and thrombin generation via procoagulants such as damage-associated molecular patterns (DAMPs), microparticles or tissue factors. Trauma also induces impairment in anticoagulant activities, which causes dysregulation of coagulation activation and promotes systemic hypercoagulation. Simultaneously, hyperfibrinolysis occurs through the expression of tissue plasminogen activator (t-PA). In the first hours after trauma, plasminogen activator inhibitor-1 (PAI-1) activity has not increased sufficiently to counteract this, and hyperfibrinolysis consumes α_2_-plasmin inhibitor (α_2_PI), which accelerates further fibrinolysis. This pattern of hypercoagulation and hyperfibrinolysis causes consumption of platelets and coagulation factors, resulting in DIC with a fibrinolytic phenotype characterized by bleeding tendency when hemostasis becomes dysregulated [3]. On the other hand, ATC is a state of activation of the anticoagulant protein C (PC) pathway caused by shock and tissue hypoperfusion, leading to hypocoagulation and subsequent fibrinolysis. Activated PC pathway also abrogates PAI-1 and increases t-PA, resulting in further hyperfibrinolysis [4, 5].

Antithrombin (AT) plays an important role in anticoagulation against intravascular thrombin formation through its ability to bind and inactivate thrombin by forming a thrombin-antithrombin complex (TAT). A decrease in AT level is well known to occur in the early phase of trauma as one of the factors impairing anticoagulation. It is especially notable that AT depletion occurs immediately after trauma [6–9]. Some reports have suggested that decreased AT levels are associated with persistent thrombin generation which could be a potential risk of subsequent thromboembolic complications in trauma patients [7, 10, 11]. Thus in the initial phase of trauma we need to manage not only bleeding tendency, but also hypercoagulation due to the impairment of the anticoagulation system in the subsequent phase. Several studies addressing sepsis or septic DIC have suggested that decreased AT levels occur due to extravascular leakage, increased AT consumption, decreased protein synthesis or degradation by enzymes released from neutrophils [12–15]. Nevertheless, the mechanism of AT depletion in trauma is still unclear. Furthermore, coagulofibrinolytic responses in patients who survive bleeding or organ damage in the early phase of trauma have not been well evaluated. Accordingly, in this study we focused on the impairment in anticoagulation that develops subsequent to the initial coagulopathy in patients who were admitted for longer than seven days as an inclusion criterion. We hypothesized that impaired anticoagulation in trauma would lead to problems even after the initial coagulopathy and organ damage had been overcome. Thus, we carried out a prospective observational study to survey dynamic changes in coagulofibrinolytic responses and to investigate the mechanisms as well as the influence of AT reduction.

## Methods

### Study design

We performed a prospective observational study collecting the data of trauma patients admitted to the tertiary Ehime University Hospital in Japan commencing in January 2015 and ending in April 2016. This study was approved by the Institutional Local Ethics Committee for Clinical Studies. Informed consent was obtained from all patients or next of kin in accordance with the Declaration of Helsinki.

### Patient selection and criteria

All adult trauma patients (≥18 years) who were admitted to our hospital either immediately following trauma or after transfer from another hospital with basically no therapeutic intervention and who were subsequently hospitalized for more than seven days were enrolled. We excluded patients who had received therapeutic interventions, including transfusion, more than 500 mL of fluid administration or medication, before admission to our hospital; those who died during initial treatment at the emergency department; those who had at least one episode of cardiac arrest; those who received anticoagulant therapy; and those who had clotting disorders such as liver cirrhosis or advanced malignancies.

Demographic data, examination findings, treatment history and mortality were recorded. Systemic inflammatory response syndrome (SIRS) was defined according to the consensus conference of the American College of Chest Physicians/Society of Critical Care Medicine [16]. Diagnoses of DIC were made based on the Japanese Association for Acute Medicine (JAAM) DIC criteria [17], by which patients were diagnosed with DIC if they had a score of 4 or higher.

The normal range of plasma AT activity is reported as 80–130% [18, 19], so we stratified the patients into two groups according to their AT levels on arrival (Day 0): normal AT group; ≥80% and lower AT group; < 80%.

### Blood sampling and measurement

Blood sampling was performed immediately upon arrival (day 0) and on days 1, 2, 4 and 6. We routinely measured blood counts and biochemistries including albumin (Alb) with TBA-c16000 (Toshiba Medical Systems, Tochigi, Japan) and XE-5000 (Sysmex, Hyogo, Japan) devices. We also used CP-2000 (Sekisui Medical, Tokyo, Japan) and STACIA (LSI Medience, Tokyo, Japan) devices to measure the biomarkers of coagulofibrinolysis, namely, prothrombin time (PT), activated partial thromboplastin time (APTT), hepaplastin test (HPT), fibrinogen (Fbg), fibrin/fibrinogen degradation product (FDP), D-dimer, thrombin-antithrombin complex (TAT), plasmin-α_2_-plasmin inhibitor complex (PIC), antithrombin (AT), protein C (PC), α_2_-plasmin inhibitor (α_2_PI) and plasminogen (PLG). After sampling, the blood samples were centrifuged at 3300 rpm for 15 min at 4 °C, and serum and plasma samples were stored at − 80 °C for subsequent analyses. We also measured total plasminogen activator inhibitor-1 (tPAI-1) on days 0, 1, 2 and 6, thrombomodulin (TM) on days 0, 2 and 6, and IL-6 on day 0 (LSI Medience). We also evaluated the development of deep vein thrombosis (DVT), which was diagnosed by ultrasonography on day 6.

### Statistical analysis

Statistical analysis was performed using the IBM SPSS 22 statistics package (IBM, Tokyo, Japan). All data are expressed as median (interquartile range: IQR) or mean ± standard deviation, as appropriate. The statistical significances of differences in patients’ clinical features, laboratory values and outcomes were assessed with Student’s t test, Mann-Whitney U test or Fisher exact test as appropriate. Time course changes of values of coagulofibrinolytic markers during the study period were tested by one-way repeated measures analysis of variance (ANOVA). The longitudinal differences in various factors between the subgroups stratified according to AT values on day 0 were analyzed by two-way repeated measures ANOVA, and pairwise comparisons were made by Student’s t test or Mann-Whitney U test as appropriate. Relationships between AT values and the other values of coagulofibrinolytic markers on day 0 were analyzed by means of linear regression analysis. A multiple regression analysis with stepwise method was applied to predict independent factors for AT levels on day 0. Covariates were selected based on coagulofibrinolytic parameters including hemoglobin, platelet, PT, APTT, HPT, Fbg, FDP, D-dimer, TAT, PIC, tPAI-1, PC, α_2_PI, PLG, TM, IL-6, Lactate acid and Alb. Variance Inflation Factor (VIF) was used to check for multicollinearity. A *p* value less than 0.05 was considered to indicate as significance.

## Results

### Patients’ clinical features and outcomes (Table 1)

Fifty-nine trauma patients were admitted to our hospital during the study period. Twenty patients were excluded according to the exclusion criteria. Thus this present study included thirty-nine trauma patients with median ISS (Injury Severity Score) of 20 (10–27), and the baseline characteristics and coagulofibrinolytic parameters on day 0 are presented in Table 1. The traumatic mechanism in all the patients was blunt injury, so most of them received several organ injuries. All of the patients had no significant medical histories such as liver cirrhosis or malnutrition which could affect coagulofibrinolytic parameters and serum albumin levels. The parameters reflecting systemic immune response and coagulofibrinolytic activation were notably elevated on day 0 (IL-6, 108.5 [40.8–250.3] pg/mL; TAT, 88.0 [30.1–200.0] μg/L; PIC, 9.1 [2.8–17.8] μg/mL). The median AT activity was 96.2 (79.8–108.3)%, which was within the normal range (80–130%) in spite of TAT elevation.

**Table 1:** Patient clinical features and outcomes

		All (*n* = 39)	AT activity on day 0		
			lower AT group AT < 80% (*n* = 10)	normal AT group AT ≥80% (*n* = 29)	*P* value
Patient characteristics					
Age	years	61 (38–73)	69 (57–80)	56 (34–72)	0.079
Sex: male / female	n (%)	26 (66.7) / 13 (33.3)	8 (80.0) / 2 (20.0)	18 (62.1) / 11 (37.9)	0.299
Injury Severity Score		20 (10–27)	26 (13–37)	17 (10–26)	0.091
JAAM DIC (+)	n (%)	11 (28.4)	6 (60.0)	5 (17.2)	0.016
Duration of injury to blood sampling					
	min	68 (31–180)	71 (42–205)	68 (30–187)	0.469
Laboratory data					
Hemoglobin	g/L	128 (118–136)	116 (91–129)	132 (120–140)	0.035
Platelet	×10^9^/L	216 (168–253)	130 (86–246)	220 (182–261)	0.033
Prothrombin time (INR)		1.07 (1.00–1.12)	1.16 (1.10–1.33)	1.03 (0.99–1.08)	< 0.001
Activated partial thromboplastin time	sec	24.7 (23.2–27.1)	27.2 (26.1–34.0)	24.3 (22.7–26.3)	0.001
Hepaplastin test	%	112.7 (91.6–126.8)	93.2 (82.1–112.4)	117.0 (96.3–127.8)	0.088
Fibrinogen	g/L	2.4 (1.9–2.8)	1.8 (1.2–2.4)	2.5 (2.3–2.8)	0.024
FDP	μg/mL	63.0 (31.4–168.1)	161.3 (100.4–292.8)	47.7 (22.6–111.7)	0.005
D-dimer	μg/mL	36.4 (16.0–96.6)	92.0 (53.5–136.5)	21.6 (10.7–59.1)	0.010
TAT	μg/L	88.0 (30.1–200.0)	99.4 (74.7–200.5)	64.0 (21.1–200.0)	0.403
PIC	μg/mL	9.1 (2.8–17.8)	15.1 (7.7–23.6)	5.8 (2.2–14.3)	0.022
tPAI-1	ng/mL	30 (3–602)	27 (19.7–113.3)	29 (16–47.5)	0.551
AT activity	%	96.2 (79.8–108.3)	69.9 (66.2–76.9)	102.5 (91.1–109.7)	< 0.001
Protein C	%	90.6 (75.8–101.5)	69.5 (54.6–83.0)	95.3 (82.1–108.8)	0.001
α_2_-plasmin inhibitor	%	90.5 (79.6–101.6)	73.8 (62.6–81.8)	95.8 (85.1–105.6)	< 0.001
Plasminogen	%	93.8 (81.5–102.6)	81.4 (64.0–88.2)	99.7 (86.9–106.7)	0.003
Thrombomodulin	ng/mL	2.9 (2.3–3.7)	3.9 (2.5–5.0)	2.9 (2.3–3.7)	0.076
Interleukin-6^a^	pg/mL	108.5 (40.8–250.3)	241.0 (145.5–411.0)	69.5 (33.0–175.5)	0.004
Lactate acid	mmol/L	1.7 (1.2–2.4)	2.2 (1.1–2.9)	1.7 (1.2–2.1)	0.281
Albumin	g/L	39 (34–43)	31 (29–36)	42 (38–43)	< 0.001
Transfusion Day 0 / Day 0–6					
Packed red blood cells	mL	0 (0–0) / 0 (0–560)	140 (0–560) / 280 (280–770)	0 (0–0) / 0 (0–0)	0.094 / 0.011
Fresh frozen plasma	mL	0 (0–480) / 0 (0–480)	480 (0–840) / 480 (0–1680)	0 (0–0) / 0 (0–0)	0.015 / 0.018
Platelets	mL	0 (0–0) / 0 (0–0)	0 (0–0) / 0 (0–100)	0 (0–0) / 0 (0–0)	0.640 / 0.014
Intervention Day 0 / Day 0–6					
Interventional radiology	n (%)	6 (15.3) / 6 (15.3)	3 (30.0) / 3 (30.0)	3 (10.3) / 3 (10.3)	0.162 / 0.162
Craniotomy	n (%)	3 (7.6) / 3 (7.6)	1 (10.0) / 1 (10.0)	2 (6.8) / 2 (6.8)	0.600 / 0.600
Laparotomy	n (%)	1 (2.5) / 1 (2.5)	1 (10.0) / 1 (10.0)	0 (0.0) / 0 (0.0)	0.256 / 0.256
Open reduction and Internal fixation	n (%)	0 (0.0) / 14 (35.9)	0 (0.0) / 4 (40.0)	0 (0.0) / 10 (34.4)	none / 0.519
Outcome					
DVT (+)	n (%)	6 (15.4)	2 (20.0)	4 (13.7)	0.490
In-hospital Mortality	n (%)	2 (5.1)	0 (0.0)	2 (6.8)	0.547

Values are presented as median (interquartile range: IQR) or number (%), if appropriate

*AT* antithrombin, *JAAM* Japanese Association for Acute Medicine, *DIC* disseminated intravascular coagulation, *FDP* fibrin/fibrinogen degradation product, *TAT* thrombin-antithrombin complex, *PIC* plasmin-α_2_-plasmin inhibitor complex, *tPAI-1* total plasminogen activator inhibitor-1, *DVT* deep vein thrombosis; n, numbers of patients

^a^measurements of 38 patients. We started to measure IL-6 from the second patient of this study

During the study period, seventeen patients of all patients (43.5%) underwent transfusion, sixteen of whom (94.1%) received transfusion on day 0. Therapeutic interventions such as interventional radiology (IVR), craniotomy, laparotomy or open reduction and internal fixation (ORIF) were performed on twenty-one patients (53.8%), ten of whom (47.6%) received these interventions on day 0. The development of DVT without associated symptoms was observed in six patients (15.3%). Two patients (5.1%) died after the study period of seven days from brain swelling due to severe head injury.

### Time course changes in coagulofibrinolytic markers (Fig. 1)

**Fig. 1:**
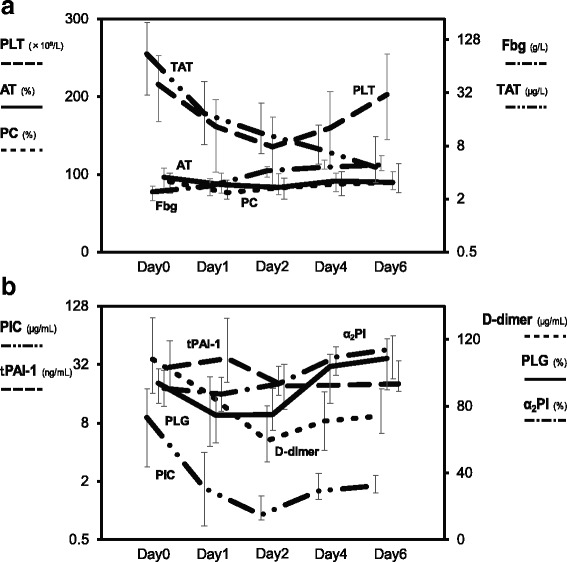
Time course of coagulofibrinolytic markers. **a**) coagulatory parameters and anticoagulants: TAT increased to the maximum level just after trauma on day 0, then remarkably decreased over time (*p* < 0.05). AT and PC slightly decreased from day 0 to 1 (*p* < 0.05). **b**) fibrinolytic parameters and inhibitors of fibrinolysis: PIC reached its maximum level on day 0, which was followed by a drastic drop to the minimum level on day 2 (*p* < 0.0001), then another increase (*p* < 0.001). α_2_PI showed a trend of decreasing from day 0 to day 1 (*p* = 0.18). tPAI-1 increased from day 0 to 1, then dropped on day 2 (*p* < 0.0001), and gradually increased along with PIC (*p* < 0.01)

The time courses of the mean values of coagulofibrinolitic biomarkers are presented in Fig. 1. *a) Coagulatory parameters:* Levels of TAT increased to their maximum levels just after trauma on day 0, then remarkably decreased over time (*p* <  0.05). *Anticoagulants:* AT and PC decreased from day 0 to 1 (AT, *p* = 0.042; PC, *p* = 0.038). *b) Fibrinolytic parameters:* PIC reached its maximum level on day 0, drastically dropped to its minimum level on day 2 (*p* <  0.0001), then significantly increased (*p* <  0.001). The time course changes of FDP were similar to those of PIC. PLG was at its minimum level on day 1 (*p* < 0.0001). *Inhibitors of fibrinolysis:* α_2_PI showed a trend of decreasing from day 0 to day 1 (*p* = 0.18). tPAI-1 increased from day 0 to 1; this change was preceded by the increases in TAT and PIC on day 0. After that, tPAI-1 dropped on day 2 (*p* <  0.0001), then gradually increased in a pattern of similar to that of PIC (*p* < 0.01).

### Correlations between AT and the other parameters including coagulofibrinolytic biomarkers on day 0 (Fig. 2 and Table 2)

**Fig. 2:**
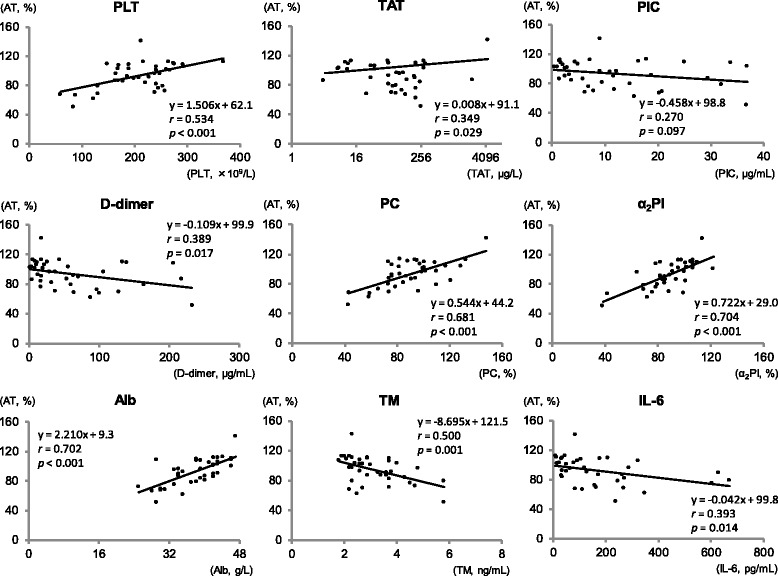
A single linear regression analysis between AT and Alb on day 0. *r*, correlation coefficient; *p*, *p* value

We performed linear regression analyses to evaluate the relationships between AT activities and the other parameters on day 0. As Fig. 2 shows, various parameters such as PLT or D-dimer showed correlations with AT activities (PLT, standard regression coefficient [*r*] = 0.534, *p* <  0.001; D-dimer, *r* = 0.349, *p* = 0.029), but TAT and PIC did not show negative linear correlations with AT (TAT, regression coefficient [*B*] = 0.008, standard error [*SE*] = 0.004, *r* = 0.349, *p* = 0.029; PIC, *p* = 0.097). There was a strong linear correlation between AT and Alb values on day 0 (*r* = 0.702, *p* < 0.001). PC and α_2_PI were also strongly correlated with AT (PC, *r* = 0.681, *p* < 0.001; α_2_PI, *r* = 0.704, *p* < 0.001). IL-6 and TM, reflecting systemic inflammatory responses and endothelial injury, respectively, also showed significant negative linear correlations with AT (IL-6, *r* = 0.394, *p* = 0.014; TM, *r* = 0.500, *p* = 0.001). A multiple linear regression analysis was performed for which the covariates were selected based on the laboratory parameters presented in Table 1. The analysis showed that Alb, α_2_PI and PC were independent predictors of AT changes on day 0 (Table 2). None of the VIF values reached as high as 10, indicating that there was no collinearity in the model.

**Table 2:** A multiple linear regression analysis for predicting AT activity on day 0 (*N* = 39)

	*B* (*SE*)	95% CI	*β*	*p*	*R* ^*2*^
Alb	1.097 (0.409)	0.266–1.928	0.349	0.011	
PC	0.286 (0.094)	0.094–0.477	0.358	0.030	0.679
α_2_PI	0.277 (0.141)	−0.010–0.564	0.270	0.058	

*B* regression coefficient, *SE* standard error, *CI* confidence interval, *β* standard regression coefficient, *p p* value, *R*^*2*^ coefficient of determination

### Comparisons of coagulofibrinolytic biomarkers depending on AT activities on day 0 (Table 1 and Fig. 3)

As shown in Table 1, ten patients showed decreased AT activity levels below the lower limit of the normal range on day 0. The median AT activity in this lower AT group (*n* = 10) was 69.9 (66.2–76.9)%, whereas that in the normal group (*n* = 29) was 102.5 (91.1–109.7)%. The lower AT group showed significantly lower AT levels as well as lower levels of PC, another anticoagulant, compared to the normal group throughout the study period.

**Fig. 3:**
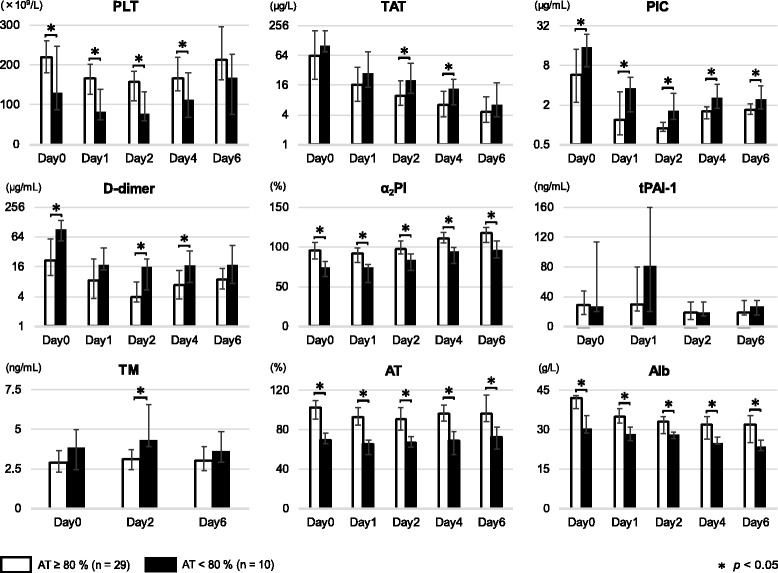
Comparisons of coagulofibrinolytic biomarkers stratified with AT activity on day 0. TAT did not show any differences on days 0 and 1, but TAT on days 2 and 4 was significantly higher in the lower AT group than in the normal group (*p* < 0.05). TM levels throughout the study period were significantly higher in the lower AT group than in the normal group (*p* < 0.05). *p < 0.05

TAT on day 0 was not significantly different between the two groups (*p* = 0.403 in Table 1). Although the rates of DVT development were not different between the two groups (Table 1), by days 2 and 4, TAT levels were significantly higher than in the normal group (*p* < 0.05, in Fig. 3), suggesting sustained intravascular coagulation.

PLT (Fig. 3) and coagulation factors (PT, Fbg, not shown in the Figure) were significantly lower in the lower AT group than in the normal group (*p* < 0.05). Regarding fibrinolytic parameters, the lower AT group exhibited a significantly greater decrease in α_2_PI and greater increases in PIC, D-dimer (Fig. 3) and FDP (not shown in the Figure) as compared to the normal group (*p* < 0.05). The frequency of transfusion during the study period was greater in the lower AT group than in the normal group (packed red blood cells, *p* = 0.011; fresh frozen plasma, *p* = 0.018; platelets, *p* = 0.014, as seen in Table 1), though there were no differences between the two groups regarding the frequencies of other treatments.

IL-6 levels on day 0 and TM levels during the study period were significantly higher in the lower AT group than in the normal group (IL-6, *p* = 0.004; TM, *p* < 0.05 in Table 1 and Fig. 3). The lower AT group showed significantly lower Alb levels than the normal group throughout the study period (*p* < 0.05 in Fig. 3).

## Discussion

It is known that the evaluation of physiological hemostatic responses to trauma through the measurement of coagulofibrinolytic biomarkers can be compromised if the measurement is taken during one of three events: fibrinolytic activation, fibrinolytic shutdown and fibrinolytic reactivation [20]. Just after a trauma insult, fibrinolytic activation occurs simultaneously with coagulatory activation. This is followed by fibrinolytic inhibition (also known as fibrinolytic shutdown) due to increasing tPAI-1, a controller for excessive fibrinolysis; fibrinolytic shutdown lasts from several to 24 h after a trauma insult or even for several days. After the repair of injured vessels and tissues, tPAI-1 decreases, reactivating fibrinolysis to allow the removal of the fibrin attached to the vessels for hemostasis. In this study, each of these three phases of physiological hemostatic response to trauma was actually observed. This shows that trauma- induced hypercoagulation and the triphasic changes of fibrinolytic activation will occur even in mild trauma patients such as those included in this study. Thus we should be aware of these time course changes to ensure the appropriate timing of treatments such as transfusions, antifibrinolytics or anticoagulants.

This study also focused on impairment in anticoagulation and decreased AT activity in the early phase of trauma. On day 0, which is thought to lie within the fibrinolytic activation phase, AT levels altered along with complex changes in coagulation factors and anticoagulants as well as fibrinolytic factors and their inhibitors. In particular, coagulofibrinolytic markers such as TAT and PIC changed simultaneously and in parallel with one another, even shortly after trauma. Nevertheless, AT did not linearly correlate with TAT and PIC on day 0, which means that, even if coagulation and fibrinolysis are activated, as evidenced by elevated TAT and PIC levels, AT levels do not decrease in response to their activation. AT inactivates thrombin’s effects by forming a covalent stable stoichiometric 1:1 complex, TAT. Thus, TAT levels directly reflect the consumption of AT against intravascular thrombin formation. Indeed, TAT value is used as a marker of coagulation activity in the diagnosis of DIC. The normal concentration of AT in human plasma is approximately 125 to 160 μg/mL, which corresponds to 80 to 130% AT activity [18, 19]. Actually, Aibiki et al. have demonstrated a very strong linear correlation between AT activity level and its concentration [15]. Regarding the association between AT and TAT, for example, even the maximum level of TAT in this study was 4385 μg/L (approximately 4.4 μg/mL), an amount much smaller than the AT levels in plasma as mentioned above. Taking these findings together, it is reasonable to assume that decreased AT levels do not result merely from AT consumption even if coagulation is activated.

In this study, changes in plasma Alb, which was one of the predictors of AT on day 0, showed a very strong linear correlation with AT. Alb, a 66-kDa protein synthesized in the liver, is well known as a parameter reflecting an impairment in liver function or extravascular leakage when vascular permeability increases resulting from acute systemic inflammatory responses [21, 22]. Our results suggest that the decrease in AT activity in the initial phase of trauma could be mainly due to systemic responses to trauma strongly associated with Alb values on day 0. Previously, the mechanisms underlying the decrease in AT during trauma have been thought to be related to increased AT consumption, decreased synthesis, extravascular leakage or degradation by enzymes released from neutrophils [3, 8, 9, 23]. However, as presented clearly in this study, AT level does not decrease merely through AT consumption. Furthermore, in this study, hemodilution through fluid administration is not likely to have caused AT concentration changes because one criterion of this study excluded patients who had been administered more than 500 mL of fluids before admission (day 0), and because the blood samples on admission were generally drawn before the initial infusion was started.

Previous studies analyzing septic or obstetrical DIC have demonstrated strong correlations between AT and Alb and revealed that one of the main causes of decreasing AT is leakage from the capillary vessels [14, 15, 24]. Although there is a possibility of impaired synthesis of AT and Alb in the liver, it is highly unlikely if not impossible that liver dysfunction would occur immediately after trauma such that it could simultaneously cause decreased AT and Alb levels just after the insult. Although we were unable to obtain measurements of Alb values in our study subjects before their trauma insults, there were no medical histories of malnutrition or comorbidities that might affect Alb levels as far as we know. On the other hand, it is well known that vascular permeability to plasma contents is restricted to around 70 kDa, so Alb (66 kDa) is mostly retained in the intravascular space under normal conditions. In inflammatory situations, however, vascular permeability increases such that even high molecular weight proteins including Alb become permeable [25]. The molecular weight of AT is 64 kDa, similar to that of Alb, so it is reasonable to presume that AT exhibits similar dynamics in extravascular leakage depending on vascular permeability. Our results clearly show that AT levels decreased immediately after trauma and that this decrease was accompanied by a very strong linear correlation with Alb. Furthermore, these decreases in AT were significantly associated with elevations of IL-6 and TM, markers of systemic inflammatory responses and endothelial injury, respectively. Thus, the present results indicate that AT could decrease due to trauma-induced systemic responses. Also, it is likely that vascular permeability strongly affects AT metabolism. The endothelial glycocalyx layer (EGL) is known as a major player in determining vascular permeability [26, 27]. EGL decreases in response to elevatied IL-6 levels during sepsis and trauma [28, 29]. Di Battista et al. have reported that endotheliopathy, which is associated with glycocalyx breakdown, occurs in the initial phase of brain injury [30]. Furthermore, Rodriguez et al. demonstrated that traumatic endotheliopathy was associated with leakage of Alb even on admission [31]. Although we explored the correlations between AT and Alb, IL-6 or TM, we did not measure any direct markers that reflect vascular permeability in this study. In the future we aim to examine the relationship between EGL and AT activity in trauma. As another plausible explanation for AT depletion after trauma, neutrophil elastase involvement is possible [3, 9], though this awaits further clarification.

Decreased AT could be a potential risk factor for subsequent thrombosis [32]. Although in this study we could not detect an association between decreased AT activity and the development of DVT, decreased AT levels were found to be associated with subsequent thrombin activation indicated by increases in TAT on days 2 and 4. A previous study in patients with AT deficiency showed a persistent elevated thrombin activation [33]. In hereditary AT deficiency, AT levels are typically 40–60% of normal levels and patients have a lifetime risk of venous thromboembolism (VTE) [18]. Furthermore, an increased risk of recurrence of VTE has been reported even in mild AT deficiency (70–80%) [34]. These reports suggest that a reduction in AT levels should be recognized as a cause of thromboembolic complications even when AT levels are not severely decreased. In trauma patients, previous studies have demonstrated systemic increases in thrombin generation in connection with depleted AT levels [7, 10], which supports the present results. Furthermore, low AT levels in trauma patients have been reported to be associated with thromboembolic complications [11]. We need to explore the necessity and the timing of anticoagulant therapy including AT supplementation with regard to vascular permeability for patients with decreased AT activity due to trauma.

### Limitations of the study

Several limitations of the present study should be addressed. Firstly, the sample size of this study was small. This means that, although we obtained the present results using the appropriate statistics, in the future we will need larger scale studies to test the hypothesis that has arisen from the present study. Secondarily, we included cases with mild trauma severity who were hospitalized for more than seven days. As one of the aims of this study was to examine time course changes in coagulofibrinolytic markers during the study period, we needed to include patients with mild severity. Yet even in patients with mild severity, we detected clear coagulofibrinolytic responses. However, further studies, including more severe cases, might be required to disclose the hemostatic conditions in different situations. Thirdly, we did not sufficiently address issues related to the trauma site, since many patients included in this study suffered from multiple organ damage due to blunt trauma. Thus there is a possibility that coagulofibrinolytic responses differ depending on the injured organs, especially if the brain is involved. Therefore, future studies must consider specific trauma sites.

## Conclusions

Coagulofibrinolytic responses occurred even in mild trauma patients who survived initial coagulopathy and organ injuries. In such situations our results showed an impairment in anticoagulation due to decreased AT activity, which could result in prolonged hypercoagulation. This is the first report examining the mechanisms of decreased AT levels in the early phase of trauma. The observed decrease in AT levels in the initial phase of trauma is unlikely to occur through AT consumption accompanied by coagulofibrinolytic activation. One important cause of decreased AT levels could be trauma-induced systemic responses such as vascular leakage as suggested by Alb depletion along with elevated IL-6 and TM.

## Abbreviations

Alb: Albumin
APTT: Activated partial thromboplastin time
AT: Antithrombin
ATC: Acute traumatic coagulopathy
DIC: Disseminated intravascular coagulation
DVT: Deep vein thrombosis
EGL: Endothelial glycocalyx layer
Fbg: Fibrinogen
FDP: Fibrin/fibrinogen degradation product
HPT: Hepaplastin test
ISS: Injury Severity Score
IVR: Interventional radiology
JAAM: the Japanese Association for Acute Medicine
ORIF: Open reduction and internal fixation
PAI-1: Plasminogen activator inhibitor-1
PC: Protein C
PIC: Plasmin-α_2_-plasmin inhibitor complex
PLG: Plasminogen
PT: Prothrombin time
SIRS: Systemic inflammatory response syndrome
TAT: Thrombin-antithrombin complex
TIC: Trauma-induced coagulopathy
TM: Thrombomodulin
t-PA: Tissue plasminogen activator
VTE: Venous thromboembolism
α_2_PI: α_2_-plasmin inhibitor
